# A new miniaturized system for extracorporeal membrane oxygenation in adult respiratory failure

**DOI:** 10.1186/cc8213

**Published:** 2009-12-17

**Authors:** Thomas Müller, Alois Philipp, Andreas Luchner, Christian Karagiannidis, Thomas Bein, Michael Hilker, Leopold Rupprecht, Julia Langgartner, Markus Zimmermann, Matthias Arlt, Jan Wenger, Christof Schmid, Günter AJ Riegger, Michael Pfeifer, Matthias Lubnow

**Affiliations:** 1 Department of Medicine II, University Hospital Regensburg, Franz-Josef-Strauss-Allee 11, 93053 Regensburg, Germany; 2 Department of Cardiothoracic Surgery, University Hospital Regensburg, Franz-Josef-Strauss-Allee 11, 93053 Regensburg, Germany; 3 Department of Anaesthesiology, University Hospital Regensburg, Franz-Josef-Strauss-Allee 11, 93053 Regensburg, Germany; 4 Department of Medicine I, University Hospital Regensburg, Franz-Josef-Strauss-Allee 11, 93053 Regensburg, Germany

## Abstract

**Introduction:**

Mortality of severe acute respiratory distress syndrome in adults is still unacceptably high. Extracorporeal membrane oxygenation (ECMO) could represent an important treatment option, if complications were reduced by new technical developments.

**Methods:**

Efficiency, side effects and outcome of treatment with a new miniaturized device for veno-venous extracorporeal gas transfer were analysed in 60 consecutive patients with life-threatening respiratory failure.

**Results:**

A rapid increase of partial pressure of arterial oxygen/fraction of inspired oxygen (PaO_2_/FiO_2_) from 64 (48 to 86) mmHg to 120 (84 to 171) mmHg and a decrease of PaCO_2 _from 63 (50 to 80) mmHg to 33 (29 to 39) mmHg were observed after start of the extracorporeal support (*P *< 0.001). Gas exchange capacity of the device averaged 155 (116 to 182) mL/min for oxygen and 210 (164 to 251) mL/min for carbon dioxide. Ventilatory parameters were reduced to a highly protective mode, allowing a fast reduction of tidal volume from 495 (401 to 570) mL to 336 (292 to 404) mL (*P *< 0.001) and of peak inspiratory pressure from 36 (32 to 40) cmH_2_O to 31 (28 to 35) cmH_2_O (*P *< 0.001). Transfusion requirements averaged 0.8 (0.4 to 1.8) units of red blood cells per day. Sixty-two percent of patients were weaned from the extracorporeal system, and 45% survived to discharge.

**Conclusions:**

Veno-venous extracorporeal membrane oxygenation with a new miniaturized device supports gas transfer effectively, allows for highly protective ventilation and is very reliable. Modern ECMO technology extends treatment opportunities in severe lung failure.

## Introduction

Despite relevant improvements in the treatment of acute respiratory distress syndrome (ARDS) mortality remains high. The estimated annual number of deaths due to acute lung injury was calculated as 74,500 for the US in a population-based study in 2005 [1]. Mortality in severe ARDS with a high lung-injury score (>3.5) and a low oxygenation index is reported to be considerably higher and may reach more than 80% [2,3]. An observational study in Europe found a mortality rate of 62.5% for ARDS with a PaO_2_/FiO_2_-ratio below 150 mmHg [4].

Extracorporeal membrane oxygenation (ECMO) has been advocated as rescue therapy in severe ARDS with presumed improved survival in specialized centres [5-7]. Historic randomised clinical trials could not prove superiority of ECMO-treatment compared to conventional treatment [8,9]. High rates of thrombo-embolic complications and hemorrhagic events had been reported, so that ECMO treatment gained only limited acceptance in adults.

Recently, a new miniaturized system for long-term extracorporeal gas exchange has been approved in Europe. The small size of this device with reduced foreign surface combined with heparin-coating, a plasma-resistant membrane and improved pump technology decreases the need for systemic anticoagulation. Encouraging results in the treatment of pediatric patients [10] and adults with cardiogenic shock [11] have been published. Hitherto, there have been no reports on the use of this system in acute lung failure. We present our experience in a relevant sample of adult patients with severe ARDS, analysing the efficiency of the device as well as reporting on observed complications and patient outcome.

## Material and methods

### Study population

From April 2006 to December 2008, 60 patients with severe lung failure were treated with the new device in a veno-venous mode. The leading cause of lung failure was pneumonia; all diagnoses are listed in Table 1. More than 70% of patients were transferred from external hospitals. According to a predefined algorithm, in all patients an attempt to improve oxygenation with conventional ventilation was undertaken. This included an effort to optimise positive end-exspiratory pressure (PEEP); one recruitment manoeuvre was done in patients with early ARDS. In patients, who were hemodynamically stable, prone positioning was attempted to optimize gas exchange. Six patients were on high-frequency oscillatory ventilation before extracorporeal support, all other patients were ventilated in a pressure-controlled mode. If stabilisation efforts were not successful after several hours and severe ARDS [12] with a PaO_2_/FiO_2 _ratio of <85 mmHg and/or severe respiratory acidosis with a pH of <7.25 persisted, extracorporeal lung support was considered. In 10 patients proving too instable for transport, our mobile team implanted the portable veno-venous ECMO in the referring hospital. Patient characteristics before inclusion are specified in Table 2. Patients on a veno-arterial ECMO were not included in this analysis.

**Table 1:** Diagnosis leading to ARDS and outcome

	All	Survival off ECMO	Survival to Discharge
**Total (No, %)**	60 (100)	37 (62)	27 (45)
**Pneumonia**	25	16	14
**Aspiration**	11	6	3
**Sepsis**	15	7	3
**Multiple Trauma**	4	3	2
**Other**	5	5	5

Other = two cases of diffuse alveolar hemorrhage, one case each of fulminant Still's disease, near fatal asthma and combined pneumonia with pulmonary embolism

**Table 2:** Patient data and characteristics before extracorporeal lung support

	All Patients	Survivors	Non-Survivors
**Number**	60	27 (45%)	33 (55%)
**Age (Years)**	53 (21 to 78)	49 (21 to 74)	54 (21 to 78) *
**Female/Male Ratio**	15/45	6/21	9/24
**BMI (kg/m^2^)**	29 (25 to 33)	27 (24 to 33)	29 (26 to 33)
**Days on Ventilation**	1.0 (1.0 to 4.8)	1.0 (0.9 to 2.0)	2.0 (1.0 to 7.0)
**SOFA Score**	14 (11 to 16)	11 (10 to 14)	15 (13 to 17) *
**LIS Score**	3.6 (3.3 to 3.8)	3.5 (3.3 to 3.8)	3.8 (3.3 to 3.8)
**Acute Renal Failure**	28 (47%)	6 (22%)	22 (67%) **
**Norepinephrine (mcg/kg/min)**	0.35 (0.15 to 0.84)	0.56 (0.15 to 0.86)	0.33 (0.16 to 0.81)
**PaO_2_/FiO_2 _(mm Hg)**	64 (48 to 86)	68 (53 to 92)	62 (48 to 86)
**PaCO_2 _(mm Hg)**	63 (50 to 80)	62 (52 to 89)	64 (47 to 74)
**pH**	7.20 (7.13 to 7.30)	7.18 (7.12 to 7.24)	7.24 (7.13 to 7.35)
**Tidal Volume (mL)**	495 (401 to 570)	489 (393 to 599)	500 (404 to 570)
**TV/kg pred. BW (mL)**	7.4 (6.1 to 8.6)	7.2 (6.1 to 8.9)	7.6 (6.1 to 8.5)
**Minute Volume (mL/min)**	13.1 (11.0 to 16.2)	13.1 (10.0 to 16.8)	13.5 (11.2 to 16.2)
**PIP (cm H_2_O)**	36 (32 to 40)	35 (31 to 39)	36 (33 to 40)
**PEEP (cm H_2_O)**	16 (13 to 20)	16 (13 to 20)	16 (14 to 20)

Data are presented as median (interquartile range) except for *female/male ratio *and *acute renal failure*. For *age*, the full range is given. * *P *< 0.05; ** *P *< 0.001

BMI = body mass index, SOFA = sequential organ failure assessment, LIS = lung injury score, TV/kg pred. BW = tidal volume per kilogram predicted bodyweight, PIP = plateau inspiratory pressure, PEEP = positive end-exspiratory pressure.

### Technique of extracorporeal support

**Figure 1:**
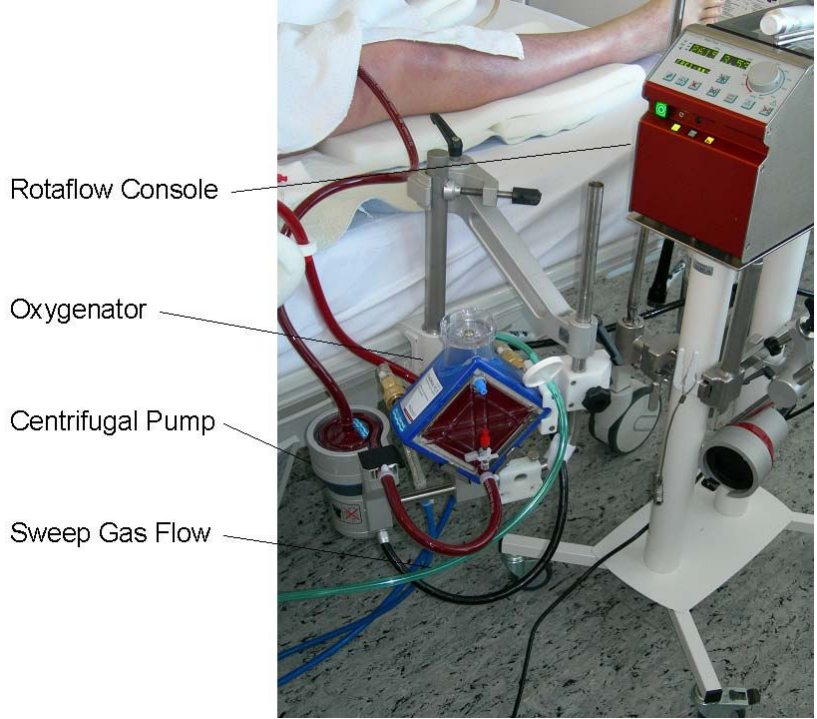
**Miniaturized device for extracorporeal lung support**. The device was obtained from Maquet Cardiopulmonary AG, Hirrlingen, Germany.

The extracorporeal system consists of two venous cannulae, a centrifugal pump and a membrane oxygenator (Figure 1). For outflow commonly the right femoral vein was cannulated in Seldinger technique with a long 21 to 23-Fr cannula (Sorin-Group Deutschland-GmbH, Munich, Germany). For reinfusion a short 15 to 17-Fr cannula (NovaLung-GmbH, Talheim, Germany) was used, that was usually implanted in the right internal jugular vein. Blood flow was generated by a centrifugal pump (Rotaflow-Centrifugal Pump, Maquet-Cardiopulmonary-AG, Hirrlingen, Germany) with an integrated battery for transport. The membrane oxygenator (PLS-Quadrox_D_, Maquet-Cardiopulmonary-AG) is made of polymethylpentene, which avoids plasma-leakage and has a total gas exchange surface of 1.8 m^2 ^with a very low inherent resistance. The filling volume of the complete device is between 400 and 500 mL, depending on the length of the tubing. The whole system is coated with heparin, hence a pronounced systemic anticoagulation is unnecessary and a partial thromboplastin time (aPTT) of 1.5 normal is sufficient. Usually 100 mg/day of acetylsalicylic acid were given to inhibit platelet aggregation. Oxygen was used as sweep-gas with a flow of 1 to 12 L/min. Blood gas analysis was done with Radiometer-700 (Radiometer, Copenhagen, Denmark).

The oxygen transfer capacity of the ECMO was calculated by multiplying the difference of oxygen content pre and post ECMO with the blood flow. The approximated carbon dioxide content, calculated by the blood gas analyser with consideration of the Henderson-Hasselbalch equation, plasma-PCO_2 _and pH, was used for calculation of carbon dioxide removal, as CO_2 _measurement in exhaust-gas, the preferable method, was not available.

### Patient management on ECMO and weaning from extracorporeal support

After implementation of the ECMO, invasiveness of ventilation was reduced to diminish further ventilator-induced lung injury (VILI). Goals for oxygenation were a PaO_2 _of 75 mmHg and PaCO_2 _was adjusted to achieve a normal pH level. Accordingly, tidal volume (TV), minute ventilation (MV), inspiratory pressure (PIP) and fraction of inspired oxygen (FiO_2_) were decreased. Positive end-expiratory pressure (PEEP) was initially not reduced to avoid atelectasis due to small TV. A recommendation for substitution was given for a hemoglobin level below 8 g/dl.

After successful treatment of the underlying disease and improvement of lung function (FiO_2 _< 0.5, PEEP <10 cmH_2_O, PIP <27 cmH_2_O), extracorporeal blood flow was stepwise reduced to 1.5 L/min. Thereafter, gas flow was tapered and finally shut off for 30 minutes. If blood gases remained stable, the ECMO system was removed and decannulation with manual compression was carried out.

### Data collection and statistical analysis

Data were collected prospectively. Blood gas analysis, ventilatory parameters, hemodynamics and vasopressor therapy were documented immediately before initiation of ECMO, after two hours, thereafter once a day and for two days after ECMO treatment.

Approval for this study was gained from the Ethics Committee of the University of Regensburg. Obtainment of informed consent was deemed by the Institutional Review Board not to be required, as the device is approved for long-term therapy and use was considered a rescue therapy in the majority of patients.

Variables are reported as median and interquartile ranges, if not told otherwise, after testing for normal distribution with the Kolmogorov-Smirnov test. Nonparametric test procedures were used for paired analysis (Wilcoxon signed-rank test) and unpaired analysis (Mann-Whitney test). A *P*-value of less than 0.05 was considered statistically significant. For statistical analysis, we used SPSS 15.0 (SPSS-Inc, Chicago, IL, USA).

## Results

### Study population

A total of 60 patients with severe respiratory insufficiency were treated with the new miniaturized device, all but two fulfilling the definition of ARDS [12]. The most common cause was double-sided pneumonia, usually community acquired or due to aspiration. Further triggers of ARDS were sepsis and multiple trauma, all reasons of respiratory failure are presented in Table 1. Thirteen patients had a platelet count of <80/nl before inclusion, and 22 patients displayed signs of disseminated intravascular coagulopathy (DIC) with an aPTT of >60 sec. All but three patients depended on norepinephrine, and 47% presented with acute renal failure, defined as need for replacement therapy. Further patient characteristics before cannulation are summarized in Table 2.

Thirty-seven patients (62%) were successfully weaned from ECMO. Of these, 10 patients died during their further course in the intensive care unit; 30-day survival was 52%, and 27 patients (45%) survived to discharge. A significant difference between non-survivors and survivors was demonstrated for age, sequential organ failure assessment (SOFA) score and acute renal failure.

### Gas exchange, hemodynamics and respiratory parameters

**Figure 2:**
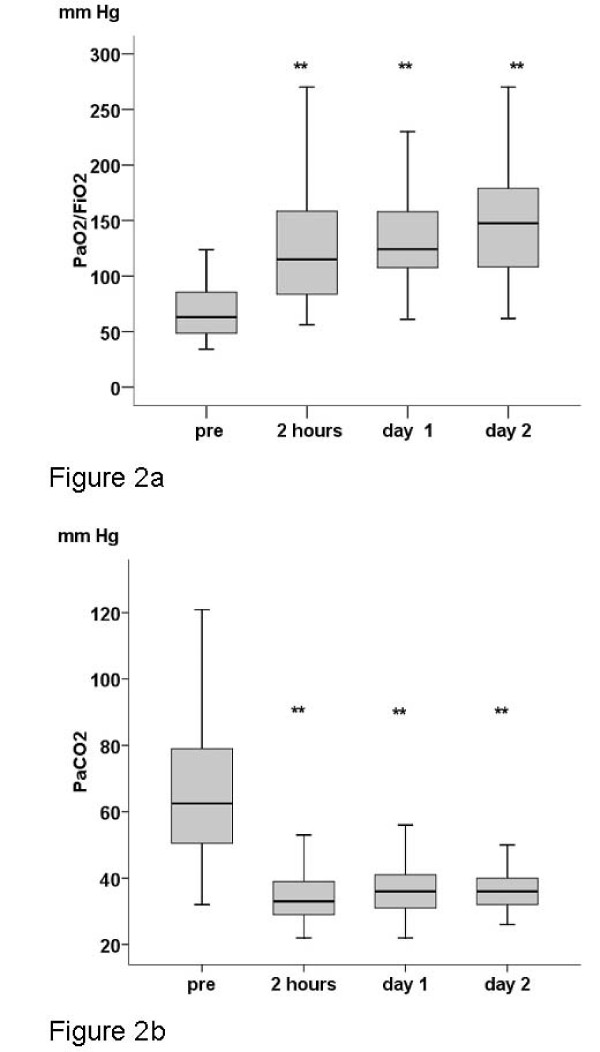
**Gas exchange parameters before and after initiation of extracorporeal support**. Figure 2a represents PaO_2_/FiO_2_, Figure 2b represents PaCO_2 _** *P *< 0.001.

Within two hours a fast rise in oxygenation occurred. PaO_2_/FiO_2 _increased from 64 (48 to 86) mmHg to 120 (84 to 171) mmHg (*P *< 0.001). In parallel, PaCO_2 _decreased from 63 (50 to 80) mmHg to 33 (29 to 39) mmHg (*P *< 0.001) (Figure 2a and 2b). Respiratory acidosis was controlled within two hours. Additional data for the following days on and after ECMO are summarized in Table 3. Simultaneously mean arterial pressure (MAP) increased from 67 (59 to 76) mmHg to 77 (69 to 86) mmHg (*P *< 0.001), despite a notable reduction in norepinephrine dose (*P *< 0.001).

**Table 3:** Gas exchange and hemodynamic parameters before, during and after extracorporeal support

	Pre ECMO	2 Hours	Day 1	Day 2	End of ECMO, Survivors	End of ECMO, Non-Survivors	Day 1 after ECMO
**PaO_2_/FiO_2 _(mm Hg)**	64 (48 to 86)	120 ** (84 to 171)	125 ** (108 to 160)	148 ** (108 to 191)	218 ** (181 to 290)	174 ** (136 to 193)	194 ** (144 to 245)
**PaCO_2 _(mm Hg)**	63 (50 to 80)	33 ** (29 to 39)	36 ** (31 to 41)	37 ** (32 to 40)	40 ** (35 to 46)	35 ** (27 to 42)	44 ** (36 to 49)
**pH**	7.20 (7.13 to 7.30)	7.42 ** (7.34 to 7.49)	7.43 ** (7.37 to 7.49)	7.44 ** (7.38 to 7.50)	7.43 ** (7.39 to 7.45)	7.31 * (7.21 to 7.41)	7.41 ** (7.32 to 7.47)
**MAP (mm Hg)**	67 (59 to 76)	77 ** (69 to 86)	72 * (66 to 79)	76 ** (70 to 87)	80 * (71 to 90)	65 (55 to 72)	80 * (70 to 86)
**Norepinephrine (μg/kg/min)**	0.35 (0.15 to 0.84)	0.28 ** (0.12 to 0.55)	0.15 ** (0.04 to 0.29)	0.10 ** (0.04 to 0.24)	0.00 ** (0.00 to 0.05)	0.27 (0.06 to 0.67)	0.00 ** (0.00 to 0.07)
**O_2 _transfer ECMO** **(mL/min)**		155 (116 to 182)	157 (128 to 178)	160 (129 to 195)	80 ** (74 to 93)	111 (99 to 160)	
**CO_2 _transfer ECMO** **(mL/min)**		210 (164 to 251)	193 * (164 to 218)	195 (158 to 232)	116 ** (96 to 150)	170 (136 to 225)	
**ECMO flow (L/min)**		2.7 (2.5 to 3.0)	2.8 (2.5 to 3.1)	2.9 * (2.5 to 3.2)	1.6 ** (1.5 to 1.8)	2.0 * (1.8 to 3.0)	

The measurement at the end of extracorporeal support is separated in survivors and non-survivors (to discharge).

Data are presented as median (interquartile range). * *P *< 0.05, ** *P *< 0.001 to first measurement. (ECMO = extracorporeal membrane oxygenation, MAP = mean arterial pressure)

Oxygen transfer by the device was calculated with 155 (116 to 182) mL/min after two hours and remained stable until the end of treatment (Table 3). The maximum measured value equalled a transfer of 284 mL oxygen/min. Approximated carbon dioxide elimination was calculated with 210 (164 to 251) mL/min two hours after implantation. Blood flow through the device averaged 2.7 (2.5 to 3.0) L/min at two hours with a mean rotational speed of 2500 r/min. Gas flow was started with 4 L/min and was set to 6 (4 to 8) L/min after adjustment for pH-level at two hours. Median length of treatment on ECMO was 9 (5 to 13) days. There was a trend towards longer treatment in non-survivors, the longest application lasting 33 days.

Mechanical ventilation before inclusion was highly invasive (Table 2). One day after initiation of the ECMO all parameters could be significantly reduced towards a more protective ventilation with an FiO_2 _of 0.6 (0.55 to 0.70), a PIP of 30 (27 to 34) cmH_2_O and a TV of 4.8 (3.6 to 6.1) mL/kg of predicted BW (Table 4). After termination of ECMO, MV and TV had to be increased as expected.

**Table 4:** Respiratory parameters before, during and after extracorporeal support

	*Pre ECMO*	*2 Hours*	*Day 1*	*Day 2*	*End of ECMO, Survivors*	*End of ECMO, Non-Survivors*	*Day 1 after ECMO*
**FiO_2_**	1.0 (1.0 to 1.0)	0.80 ** (0.60 to 0.90)	0.60 ** (0.55 to 0.70)	0.50 ** (0.45 to 0.68)	0.40 ** (0.35 to 0.45)	0.50 ** (0.45 to 0.60)	0.45 ** (0.40 to 0.50)
**Minute Ventilation (L/min)**	13.1 (11.0 to 16.2)	7.6 ** (4.9 to 10.0)	7.0 ** (5.0 to 8.5)	6.5 ** (4.7 to 8.5)	7.6 ** (6.2 to 11.3)	7.9 ** (5.7 to 9.8)	11.0 (10.0 to 14.0)
**Tidal Volume (mL)**	495 (401 to 570)	336 ** (292 to 404)	308 ** (254 to 400)	327 ** (229 to 415)	431 (322 to 509)	400 (293 to 497)	526 (443 to 640)
**Tidal Volume per kg pred. BW (ml/kg)**	7.4 (6.1 to 8.6)	5.1 ** (4.4 to 5.9)	4.8 ** (3.6 to 6.1)	4.8 ** (3.4 to 6.1)	6.6 (4.6 to 7.7)	5.9 (4.5 to 8.1)	7.4 (6.6 to 9.4)
**Peak Inspiratory pressure (cm H_2_O)**	36 (32 to 40)	31 ** (28 to 35)	30 ** (27 to 34)	29 ** (26 to 32)	23 ** (19 to 26)	28 ** (24 to 32)	26 ** (22 to 27)

The measurement at the end of extracorporeal support is separated in survivors and non-survivors (to discharge).

Data are presented as median (interquartile range). ** *P *< 0.001 to *pre ECMO*. (ECMO = extracorporeal membrane oxygenation, BW = body weight)

### Complications and side effects

Laboratory values before, during and after extracorporeal assist are presented in Table 5. Hemoglobin levels and platelet counts dropped during treatment with ECMO (*P *< 0.001). For every patient 9 (3 to15) red blood cell (RBC) concentrates, 4 (0 to 14) units of fresh frozen plasma (FFP) and 0 (0 to 4) platelet concentrates were transfused during the period on ECMO. The number of substituted blood products per day is displayed in Table 6 with a higher need for transfusion in non-survivors.

**Table 5:** Laboratory variables before, during and after extracorporeal support

	Pre ECMO	Day 1	Day 2	End of ECMO, Survivors	End of ECMO, Non-Survivors	Day 1 after ECMO
**Hemoglobin (g/dL)**	10.4 (9.0 to 12.3)	9.5 ** (8.8 to10.4)	9.2 ** (8.4 to 9.6)	8.7 ** (8.3 to 9.4)	8.7 * (7.7 to 9.3)	8.8 ** (8.3 to 9.9)
**Platelets (/nL)**	158 (82 to 266)	115 ** (60 to 160)	85 ** (40 to 149)	113 ** (76 to 146)	46 ** (22 to 86)	110 ** (66 to 154)
**aPTT (sec)**	53 (40 to 69)	58 (46 to 74)	53 (45 to 59)	47 (41 to 59)	62 (44 to 118)	42 (34 to 51)
**LDH (U/L)**	400 (289 to 684)	462 (293 to 885)	550 * (323 to 941)	422 (276 to 550)	903 * (442 to 9712)	444 (346 to 515)
**Lactic Acid (mg/dl)**	50 (19 to 80)	37 (19 to 73)	25 * (17 to 43)	12 ** (10 to 18)	86 (15 to 161)	11 ** (7 to 4)

The measurement at the end of extracorporeal support is separated in survivors and non-survivors (to discharge).

Data are presented as median (interquartile range). * *P *< 0.05, ** *P *< 0.001 to *pre ECMO*. ECMO = extracorporeal membrane oxygenation, LDH = lactic dehydrogenase, aPTT = activated partial thromboplastin time.

**Table 6:** Frequency of complications during treatment with ECMO

Complications	All Patients	Survivors	Non-Survivors
**Thrombosis of Oxygenator**	10	4	6
**Failure of Pump Head**	1	1	0
**Cannulation Site Bleeding**	2	2	0
**Surgical Site Bleeding**	11	4	7
**Pulmonary Hemorrhage**	3	1	2
**Diffuse Bleeding**	5	1	4
**Femoral Vein Thrombosis**	5	3	2
**Transfusion Requirements**			
			
**RBC/Day on ECMO**	0.8 (0.4 to 1.8)	0.6 (0.3 to 1.8)	1.0 (0.7 to 2.1) *
**FFP/Day on ECMO**	0.5 (0.0 to 2.6)	0.0 (0.0 to 1.5)	1.1 (0.1 to 3.1)*
**PC/Day on ECMO**	0.0 (0.0 to 0.7)	0.0 (0.0 to 0.6)	0.1 (0.0 to 0.9)

Data are presented as numbers or median (interquartile range). * *P *< 0.05.

(RBC = red blood cell concentrate, ECMO = extracorporeal membrane oxygenation, FFP = fresh frozen plasma, PC = platelet concentrate)

Complications during ECMO treatment are summarized in Table 6. Technical problems that could not be controlled were not observed. The pump head was changed due to a small thrombus in one patient with heparin-induced thrombocytopenia. A change of the oxygenator was necessary in 10 patients, which results in an average oxygenator time of eight days. An accidental dislocation of the backflow cannula happened two times, leading to rapid asystole due to hypoxia in one case. Two patients needed resuscitation during implantation; both were stabilized. One patient was converted to veno-arterial ECMO for cardiac reasons.

The most common cause for death was intractable septic shock and multiple organ failure (81%), generally despite sufficient oxygenation with ECMO, as can be seen in Table 3 (end of ECMO, non-survivors). Three patients died as a result of cardiac failure, and two patients died due to pulmonary and cerebral hemorrhage, the latter 32 days after weaning.

## Discussion

The current study presents for the first time the use of a new miniaturized system for extracorporeal membrane oxygenation in a large adult study population with severe ARDS. Crucial technical innovations, easy application and a limited number of side effects support its employment as a highly effective method to secure vital gas exchange and to reduce further VILI also in patients with an increased risk of hemorrhage.

Lately, a meta-analysis suggested that mortality from ARDS may not have decreased since 1994 [13]. Hence, the authors emphasized the need for future effective therapeutic interventions. ECMO with improved technical equipment may prove to be a valuable option. Its first successful application in a trauma patient was published in 1972 [14]. Despite disappointing outcomes in early trials [8,9], major improvements have been achieved in the following years [5-7,15-18]. Recently a randomised multicentre trial has been published, which found a significantly improved survival without severe disability in the ECMO group [19].

The current study population had a weaning rate from ECMO of 62% and a survival rate of 45%. The median age of 53 years of our study population was considerably higher compared to all former trials including the CESAR trial (Table 7). Age is known to be a risk factor for mortality [20,21]. Most of our patients were in septic shock and presented with a median SOFA-Score of 14 predicting a high mortality [22]; thrombocytopenia or DIC, traditionally exclusion criteria for ECMO, were no contraindication for inclusion. Therefore, in comparison to earlier studies our results appear acceptable in a population with a low probability for survival. Selecting the age group between 20 and 40 years (n = 14), presenting with a PaO_2_/FiO_2 _of 63 mmHg, a LIS of 3.75 and a SOFA-score of 12.5, the survival rate in our study was 79%. In a retrospective analysis, older age, acute renal failure before initiation of ECMO therapy and a higher SOFA-score were predictors of a higher mortality (Table 2). Extrapulmonary sepsis and aspiration pneumonia held a lower survival rate than double-sided pneumonia of bacterial or viral origin (Table 1).

**Table 7:** Studies and registries about ECMO in adult patients with severe ARDS

Study (Year)	Number	Age (Years)	PaO_2_/FiO_2 _(mm Hg)	LIS	Survival (%)
**CESAR Trial** **(2009) **[19].	90/68 treated	39.9 +/- 13	75.9 +/- 29.5	3.5 +/- 0.6	63
**ELSO Registry** **(2009) **[20].	1473	34 (16.0 to 84.2)	57 (46,75)	nr	50
**Beiderlinden et al** **(2006) **[18].	32	42.2 +/- 13	63 +/- 28	3.8 +/- 0.3	53
**Hemmila et al** **(2004) **[7].	255	38.4	55 +/- 16	nr	52
**Mols et al** **(2000) **[16].	62	35 +/- 11	96 +/- 51	3.2 +/- 0.4	55
**Linden et al** **(2000) **[17].	17	33.8	46	3.5	76
**Peek et al** **(1997) **[6].	50	30.1	65 +/- 37	3.4	63
**Lewandowski et al** **(1997) **[5].	49	31.5 +/- 14	67 +/- 28	3.4 +/- 0.3	55
**Morris et al** **(1994) **[9].	21	33 +/- 3.1	62.6 +/- 4.2	nr	33
**Gattinoni et al** **(1986) **[15].	43	26 (2 to 56)	62 - 72	nr	49

ELSO = Extracorporeal Life Support Organization, LIS = lung injury score, nr = not reported.

Initiation of ECMO led to a rapid and sustained improvement of blood gases and a correction of respiratory acidosis with hemodynamic stabilisation The median calculated oxygen transfer of the device amounted to 155 mL/min, which is about half of the average total oxygen consumption, as we have shown previously in a comparable group of patients with ARDS [23]. The oxygen transfer rate depends on hemoglobin, venous saturation and blood flow. As the flow rate is limited by the cannula size, many authors favour a high hemoglobin content to increase oxygen transport capacity [5,6,17]. However, several trials have shown in the past that a liberal RBC substitution may increase mortality in intensive care [24,25]. Taking both positions into account we had decided to aim at a more restrictive transfusion policy with a recommended hemoglobin level of >8 g/dl. Being aware that this reduces the oxygen transfer capacity of the device we assent that the optimal hemoglobin level for extracorporeal support is still a matter of discussion.

Carbon dioxide elimination of the new device exceeds the rate of oxygen transfer. Gattinoni proved more than 20 years ago, that carbon dioxide removal is possible with an extracorporeal flow of <30% of cardiac output [15]. Extracorporeal gas transfer allows a reduction of aggressive ventilatory patterns, which is essential to avoid further VILI. In the current study, FiO_2_, PIP and MV were reduced significantly after commencement of ECMO. TV/kg of predicted BW was decreased to 4.8 mL at Day 1, a range that can be considered highly protective [26]. In contrast to earlier studies [6,7,15,16], in the present study PEEP initially was not reduced to avoid progressive atelectasis and to preserve function of the native lung. Consequently, we did not have to provide total oxygen requirements and were able to run the ECMO in a less injurious mode.

ECMO is a procedure with potentially serious complications. With the new device, we did not encounter mechanical complications that were life threatening. In particular, rupture of the tubing or leakage of the oxygenator did not occur. Oxygenator failure was exclusively a result of slowly progressing thrombotic occlusion. As the system is coated with heparin, we aimed at an aPTT of about 1.5 normal. This decreased the rate of blood transfusions, which had been considerably higher in earlier studies [6-9], despite the fact that the current study included patients with multiple trauma, thrombocytopenia and DIC. In several patients with manifest bleeding, systemic anticoagulation was temporarily interrupted up to several days without clotting of the device. Adding all blood components together, a total of 1,777 units were given. Butch reported a transfusion need of more than 15,000 units of blood components in 74 adult patients treated with ECMO [27]. Ang found an average daily need of two units of RBC, three units of platelets and 0.6 units of FFP [28].

Peek et al recommended the transfer of adult patients with severe respiratory failure to a centre with an ECMO-based management protocol [19]. However, transport on ECMO had not been possible in the CESAR trial and five patients in the ECMO group died before transport or in transit. With the miniaturized device of the present study, interhospital transport has been carried out in 10 of our patients and others without complications [29]. Costs for the device including cannulation are estimated to be about 3,000 €. Labour resources are not high, amounting in our institution to a circuit check twice daily to document gas exchange and pressure drop across the oxygenator.

The present study has limitations. It is a single centre experience without a randomised control group. Comparison of mortality with a historical control group is biased, as general treatment of ARDS has changed substantially in the last decade. In the past mortality rates of >80% have been reported in severe ARDS [2]. A prospective randomised trial on ECMO is difficult to conduct, as many centres would consider it unethical to withhold a potentially life-saving treatment for a control group. However, with a here documented low rate of complications, a randomised prospective multicenter trial with the new device should be taken into account. This could include patients with early ARDS due to severe community-acquired pneumonia with a PaO_2_/FiO_2 _ratio <100 mmHg on optimal protective ventilation despite a trial of prone positioning. Ultra-protective ventilation on ECMO would be compared to a control group on conventional protective ventilation; in life-threatening hypoxemia (PaO_2_/FiO_2 _ratio <50 mmHg) cross-over to the ECMO group would be allowed. Several questions remain unanswered. As mentioned before, the ideal hemoglobin-content on ECMO-treatment is currently not known. As ECMO can activate inflammation and coagulation cascades, a continuing effort to optimise biocompatibility is desirable.

## Conclusions

The current miniaturized system enables extracorporeal lung support with more than 50% of total gas exchange. Improved oxygenator, tubing and centrifugal pump allow less systemic anticoagulation compared to earlier trials. The rate of hemorrhagic complications is markedly reduced, which makes its implementation possible in patients with a risk of bleeding and older age, which had been traditionally contraindications for ECMO therapy. A fast and sustained rise in PaO_2_/FiO_2 _as well as a rapid decrease in PaCO_2 _and normalization of pH resulted in a clearly more protective ventilation. Therefore, a reduction of VILI can be assumed and time was gained for lung healing. Labour resources are low, and serious technical complications were not encountered. Miniaturization and improved biocompatibility of ECMO will extend the indication for its employment from a rescue therapy at present to an effective therapeutic intervention to avoid injurious ventilator settings in ARDS in the future.

## Key messages

• The new miniaturized system for extracorporeal membrane oxygenation supports gas transfer in severe acute lung failure very effectively.

• Due to crucial technical developments the need for systemic anticoagulation is lowered, allowing application in patients with a risk of hemorrhage.

• Injurious ventilatory settings can be reduced rapidly to a highly protective mode.

• Interhospital transport is possible without major effort.

• Despite a low probability for survival, 62% of patients were weaned from the extracorporeal system, and 45% survived to discharge.

## Abbreviations

ALI: acute lung injury; aPTT: activated partial thromboplastin time; ARDS: acute respiratory distress syndrome; BMI: body mass index; BW: body weight; CESAR: Conventional ventilation or ECMO for Severe Adult Respiratory Failure; DIC: disseminated intravascular coagulopathy; ECMO: extracorporeal membrane oxygenation; ELSO: Extracorporeal Life Support Organization; FFP: fresh frozen plasma; FiO_2_: fraction of inspired oxygen; LDH: lactic dehydrogenase; LIS: lung injury score; MAP: mean arterial pressure; MV: minute ventilation; PaCO_2_: partial pressure of arterial carbon dioxide; PaO_2_: partial pressure of arterial oxygen; PC: platelet concentrate; PEEP: positive end-exspiratory pressure; PIP: plateau inspiratory pressure; RBC: red blood cell concentrate; SOFA: sequential organ failure assessment; TV: tidal volume; VILI: ventilator induced lung injury.

## Competing interests

Dr. Müller has received lecture honoraria from Maquet CardioPulmonary Care, Germany. Mr. Philipp is Chief Perfusionist at the University Hospital Regensburg and a member of the technical advisory board of Maquet CardioPulmonary Care. Prof. Schmid has no conflicts of interest to disclose, but participated in speaking activities about va-ECMO. The remaining authors declare that they have no competing interests.

## Authors' contributions

TM has made important and substantial contributions to design, acquisition of data, analysis and interpretation of data and drafted the manuscript. AP, AL, CK, TB, LR, JL and ML have made substantial contributions to treatment of patients, acquisition of data, interpretation of data and revised the manuscript critically. JW has made substantial contribution to acquisition of data and analysis of data. MH, MZ, MA, CS, GR and MP have made substantial contribution to conception and design and interpretation of data.

All authors revised the submitted article critically for important intellectual content and approved the final version.
